# Impact of Genetic Polymorphisms on Electrochemical Parameters and Acid-Base Disorders in Brazilian Runners During a 105-Kilometer Ultramarathon

**DOI:** 10.3390/nu16223945

**Published:** 2024-11-19

**Authors:** Marcelo Romanovitch Ribas, Fábio Kurt Schneider, Danieli Isabel Romanovitch Ribas, Georgian Badicu, Ana Claudia Bonatto, Luca Paolo Ardigò, Júlio Cesar Bassan

**Affiliations:** 1 Postgraduate Program in Electrical Engineering and Industrial Informatics, Universidade Tecnológica Federal do Paraná, Curitiba 80230901, Brazil; mromanovitch@yahoo.com.br (M.R.R.); fabioks@utfpr.edu.br (F.K.S.); 2 Human Genetics Laboratory, Centro Universitário Autônomo do Brasil (UniBrasil), Curitiba 82821020, Brazil; danieliribas@yahoo.com.br; 3 Department of Physical Education and Special Motricity, Faculty of Physical Education and Mountain Sports, Transilvania University of Braşov, 500068 Braşov, Romania; 4 Postgraduate Program in Physical Education, Universidade Tecnológica Federal do Paraná, Curitiba 81310900, Brazil; anacbonatto@yahoo.com.br (A.C.B.); jcbassan@utfpr.edu.br (J.C.B.); 5 Department of Teacher Education, NLA University College, 0166 Oslo, Norway

**Keywords:** polymorphism, genetic, athletic performance, marathon, running

## Abstract

Background/Objectives: This study focused on a group of 22 elite male mountain runners from Brazil (average age of 35.9 ± 6.5 years) with the objective of exploring the possible roles of the ACTN3 R577X, ACE I/D, and CK MM A/G NcoI genetic variants in shaping electrochemical profiles and maintaining acid-base homeostasis during a 105-km ultramarathon. Methods: Genotyping for each polymorphism (*ACTN3*: RR, RX, XX; *ACE*: DD, ID, II; *CK* MM: AA, AG, GG) was conducted using PCR-RFLP (Polymerase Chain Reaction-Restriction Fragment Length Polymorphism), and saliva samples were used to obtain DNA. Analyses of electrochemical and acid-base disturbances were performed in real time. Results: It was observed that athletes who completed the race in less time had lower calcium concentrations (Rs = 0.35; *p* = 0.016). Pre-race, the RX genotype showed a 14.19% reduction in potassium levels compared to RR (*p* = 0.01). The GG genotype showed potassium levels 19.36% higher than AA (*p* = 0.01) and a 6.11% increase in hematocrit values compared to AA (*p* = 0.03). Additionally, the AG genotype exhibited hematocrit values 5.44% higher than AA (*p* = 0.03). Post-race, the XX genotype demonstrated higher hematocrit values compared to RX, with an increase of 8.92% (*p* = 0.03). The II genotype showed a 0.27% increase in pH compared to ID (*p* = 0.02) and a 20.42% reduction in carbon dioxide levels (*p* = 0.01). Conclusions: The findings emphasize the impact of the examined polymorphisms on the modulation of electrochemical factors and the maintenance of acid-base equilibrium in athletes during 105 km ultramarathons.

## 1. Introduction

Mountain ultramarathons are competitions that exceed the distance of a traditional marathon (42.195 km) and involve substantial cumulative elevation gains, reaching up to 25,000 m [1]. These events, which typically last at least 10 h, pose a significant physiological challenge to the body, requiring extreme adaptations to withstand the harsh conditions [2]. Over the past three decades, the growing popularity of these races has driven research aimed at better understanding the physiological adaptations involved in long-duration events [3,4].

However, sports performance is a multifactorial trait [5]. Depending on the type of sport, approximately 66% of an athlete’s physical abilities can be explained by genetic inheritance [6]. There is considerable evidence that genetic variants, including polymorphisms in the Alpha Actinin 3 gene (*ACTN3* R577X), the Angiotensin-Converting Enzyme (*ACE* I/D), and the Creatine Kinase (*CK*M) gene (formerly known as *CK* MM, with “MM” indicating the muscle-specific isoform), play a role in affecting human muscle performance and metabolic processes [5,7].

The alpha-actinin-3 protein, which is exclusively found in fast-twitch muscle fibers, is produced by the *ACTN3* gene. This protein is essential for preserving the skeletal muscles’ shape and functionality. The R577X polymorphism is one of the genetic variants in the *ACTN3* gene that can affect the composition of muscle fibers and, in turn, athletic performance [8]. Furthermore, the R allele of the *ACTN3* R577X polymorphism is linked to strength and power sports, which are essential for activities requiring high intensity and continuous effort, such as mountain ultramarathons, whereas the X allele is more common among endurance athletes [9].

By means of the ren-in-angiotensin-aldosterone pathway, the *ACE* gene controls blood pressure [10]. Regarding the *ACE* I/D polymorphism, endurance athletes are more likely to have the I allele, which is linked to a greater percentage of type I muscle fibers [11]. The D allele, on the other hand, is linked to strength and power sports and is linked to elevated *ACE* levels, which can impact cardiovascular function while exercising [2]. Last but not least, athletes who specialize in strength and power sports are more likely to have allele G of the *CK* MM A/G Ncol polymorphism, whereas endurance athletes have a higher prevalence of allele A. It is crucial to emphasize that the *CK* MM gene contributes to the synthesis of muscular energy, and variations in this gene can impact energy [12].

The roles of the *ACTN3*, *ACE*, and *CK* MM genes in electrochemical parameters and acid-base balance are linked to their involvement in essential physiological processes. The *ACTN3* gene affects not only the composition of muscle fibers but also plays a role in muscle response and the maintenance of ionic equilibrium during extended physical exertion [8]. The *ACE* I/D polymorphism regulates angiotensin-converting enzyme activity, affecting electrolyte homeostasis and vascular response, which can alter the body’s ability to maintain pH and acid-base balance during endurance activities [10]. Similarly, the *CK* MM gene, involved in muscle energy production, plays an important role in ATP resynthesis and hydrogen ion buffering, essential mechanisms for preventing metabolic acidosis and maintaining muscle function during long-duration exercises [12]. These associations suggest that genetic variations in these loci may impact the regulation of electrochemical variables and pH stability, critical factors for performance in mountain ultramarathons.

Despite this knowledge, it is still unknown if these genes and their polymorphisms play any role in the modulation of chemical changes and acid-base disorders that interfere with performance in mountain ultramarathon events. According to a single study [2], the *ACE* I/D polymorphism did not alter the lipid profiles of C-reactive protein and interleukin 6 in 24 ultramarathon runners one week after a 100-km race. Therefore, the objective of this study is to explore the possible roles of the *ACTN3* R577X, *ACE* I/D, and *CK* MM A/G NcoI genetic variants in shaping electrochemical profiles and maintaining acid-base homeostasis in mountain runners during a 105-km ultramarathon. The primary hypothesis of this study was that the variations in the *ACTN3* R577X, *ACE* I/D, and *CK* MM A/G NcoI genes were associated with the regulation of electrochemical parameters and acid-base imbalances in long-distance mountain runners participating in 105 km races. This study addresses a significant gap in the current literature by exploring the genetic influences on physiological responses in extreme endurance events, providing insights that could enhance athlete training and performance strategies in such demanding sports.

## 2. Materials and Methods

The study involved 22 elite male athletes of European ancestry who participated in a 105 km mountain competition. The average age of the participants was 35.9 ± 6.5 years, with an average pre-race mass of 72.5 ± 6.6 kg and a body fat percentage of 10.9 ± 2.3%, measured using bioelectrical impedance analysis. After the race, the average mass was 70.8 ± 6.1 kg, with a body fat percentage of 9.3 ± 1.7%. To be eligible for the study, athletes were required to meet the following criteria: having completed at least two races over 50 km and one race over 80 km between 2015 and 2016, being free from chronic non-communicable degenerative diseases, and providing a cardiopulmonary assessment certified by a cardiologist to evaluate cardiovascular function. Athletes were excluded if they chose to withdraw their consent, did not sign the informed consent form, or failed to complete one of the two data collection stages. The athletes were considered elite due to their participation in national competitions in their respective countries and international competitions, demonstrating their skill on a global stage. These athletes have undergone rigorous training regimens and possess a higher level of physical conditioning, making them ideal candidates to investigate the nuances of genetic variations and their impact on endurance capabilities [13]. The research was approved by the local Research Ethics Committee, under CEP number 2.275.040. All study procedures were conducted in accordance with Resolution 466/12 of the Brazilian National Health Council. Data collection took place during the Ultramaratona dos Perdidos SkyMarathon^®^, held at Morro dos Perdidos in Tijucas do Sul, Paraná, and following the Declaration of Helsinki.

Using the finger-pulp puncture technique, venous blood samples with real-time access and analysis were collected from capillary blood. The skin was sanitised with 70% alcohol. The blood sample was collected using a capillary tube with a capacity of 200 μL (Capillary Tubes 250, F. Hoffmann-La Roche Ltd., Basel, Switzerland), treated with heparin. The gasometry equipment GEM Premier 3000 (Instrumentation Laboratory, Bedford, MA, USA) was used to perform blood analysis. hydrogen ion concentration (pH), partial CO_2_ pressure (pCO_2_), partial O_2_ pressure (pO_2_), Na^+^ (Sodium), K^+^ (Potassium), Ca^2+^ (Calcium), Glu (glucose), Lac (lactate), hematocrit (Hct) and HCO_3_^−^ (Sodium Bicarbonate), important variables to measure basic acid disorder, were evaluated. Blood samples were processed immediately after collection, ensuring fast and reliable results, obtained in approximately 85 s after each sample was introduced into the blood gas analyser.

Genomic DNA was extracted from all athlete samples using a phenol-chloroform extraction method. Saliva was collected by scraping the buccal mucosa, followed by a rinse with a 3% glucose solution. This approach was chosen because it is less invasive and more convenient for athletes, particularly during sports events [14]. For two days, all saliva samples were kept in a freezer at −20 °C [15]. To ensure the validity of the genotypes, a subset of samples was genotyped again following the same protocol described. No additional methods, such as QPCR or sequencing, were used to confirm the genotypes. Representative gel electrophoresis images were captured and are available as visual support for the results. Specific primers were used for the genotyping of the *ACTN3* R577X polymorphism. Details of the PCR cycling conditions can be found in Table 1, which now includes an additional column describing the number of cycles, denaturation, annealing, extension temperatures, and times. The Polymerase Chain Reaction (PCR) amplification protocol consisted of the following steps: (a) 95 °C for 5 min, (b) 94 °C for 30 s, (c) 58 °C for 30 s, (d) 72 °C for 30 s, (e) repeating steps ‘b’ through ‘d’ for 30 cycles, and (f) 72 °C for 5 min. Following amplification, the PCR product was digested with the DdeI restriction enzyme (Sigma-Aldrich, Merck KGaA, Darmstadt, Germany) at 37 °C for 4 h. The RX heterozygous variant yielded fragments of 205, 108, 97, and 86 bp; digestion of the homozygous RR allele produced fragments of 205 and 86 bp, while digestion of the homozygous mutant XX allele, which lacks *ACTN3* expression, resulted in fragments of 108, 97, and 86 bp [15].

Specific primers were employed to genotype the *ACE* I/D polymorphism (Table 1). The PCR amplification protocol consisted of: (a) an initial denaturation and enzyme activation step at 95 °C for 5 min, (b) 30 cycles of denaturation at 94 °C for 30 s, (c) annealing at 57 °C for 1 min, (d) extension at 72 °C for 1 min, and (e) a final extension at 72 °C for 5 min. The homozygous DD deletion allele of the ACE gene generated a 191 base pair fragment. In contrast, the homozygous insertion II allele generated a 478 base pair fragment, containing the 287 bp insert and the ID heterozygous allele generated fragments of 191 and 478 bp [15]. To improve genotyping specificity, samples identified with the DD genotype were re-assessed using an insertion-specific forward primer (Table 1). The PCR amplification protocol consisted of: (a) initial denaturation and enzyme activation at 95 °C for 5 min, (b) 35 cycles of denaturation at 94 °C for 30 s, (c) annealing at 56 °C for 1 min, (d) extension at 72 °C for 1 min, and following the 35 cycles, a final extension at 72 °C for 5 min. The presence of a 408 base pair band confirms the I allele, meaning samples previously genotyped as DD are now reclassified as ID. Samples initially identified as ID or II in the first reaction served as a positive control for the insertion-specific reaction [15].

Specific primers were employed to genotype the *CK* MM A/G Ncol polymorphism (Table 1). The PCR amplification protocol followed these steps: (a) an initial denaturation cycle at 95 °C for 5 min, (b) 35 cycles of denaturation at 95 °C for 30 s, (c) annealing at 66 °C for 30 s, (d) extension at 72 °C for 1 min, and (e) a final elongation step at 72 °C for 10 min. Following amplification, the PCR product was digested with the Ncol restriction enzyme (New England Biolabs, Ipswich, MA, USA) under specified conditions. The allele lacking the Ncol restriction site, identified as the homozygous GG allele, produced a 1170 bp fragment (mutant, rare), whereas the allele with the Ncol polymorphic site, designated as the homozygous AA allele, generated fragments of 985 and 185 bp (wild type). The heterozygous AG allele resulted in fragments of 1170, 985, and 185 bp [15]. For genotype analysis, electrophoresis (Kasvi, Pinhais, Brazil) was conducted on a 3% agarose gel, stained with ethidium bromide, and visualized using a UV transilluminator [15]. All data were analyzed using descriptive statistics (mean, standard deviation, and percentages) with R software version 4.0.5 (https://cran.r-project.org/bin/windows/base/old/4.0.5/, accessed on 30 March 2022). Sample size calculations were performed using the GPower software (version 3.1.9.2, Heinrich-Heine-Universität Düsseldorf, Düsseldorf, Germany), based on a standardized difference of 0.7, with α = 0.05 and power = 0.8 as significance criteria. Power analysis indicated that a total sample size of 19 participants would be required to detect a significant difference in measurements. Genotype distribution for the *ACTN3* R577X, *ACE* I/D, and *CK* MM A/G NcoI polymorphisms in the general population was tested using Hardy–Weinberg equilibrium. Comparisons of genotype distribution and allele frequencies were conducted using Pearson’s Chi-square test, with Yates correction applied when frequencies were below five. Effect size between groups was measured using Cramer’s V. Bonferroni correction was applied to *p*-values to account for multiple comparisons. To investigate the relationship between genotypes of the studied polymorphisms and running times, as well as the impact of these genotypes on electrochemical variables and acid-base disturbances, normality was first assessed using the Shapiro–Wilk test, followed by an independent samples t-test. Effect size between groups was calculated using Hedges’ g with a 95% confidence interval (CI). Correlations between electrochemical elements, acid-base disturbances, and running time were analyzed using the nonparametric Spearman correlation coefficient (Rs). A significance level of *p* ≤ 0.05 was used for all statistical analyses.

### Prior Publication of Genotyping Data

The genotyping data were previously analyzed and published in the study by Ribas et al. (2023) [15], which focused on the relationship between genetic polymorphisms and body composition. In the present study, however, the focus is distinct, addressing electrochemical variables and acid-base disturbances before and after a 105 km race. This section was included to ensure transparency and clarify that the current manuscript differs in approach and objectives.

## 3. Results

The genotypic distribution and allele frequencies of the *ACTN3* R577X, *ACE* I/D, and *CK* MM A/G Ncol polymorphisms are presented in Table 2. When analysing these polymorphisms, we observed a predominance of heterozygous genotypes associated with strength, power and endurance (RX, ID and AG) among the participants (RX = 54.4%, ID = 63.6% and AG = 50%, χ2 with Yates correction = 7.23, with *p* = 0.0001, *p* = 0.0001, and *p* = 0.007, in that order; adjusted *p*-values: 0.001, 0.001, and 0.07). Genotypes RX, ID and AG exhibited a high effect size (1.12, 0.98 and 0.62). The genotypic distribution is in Hardy–Weinberg equilibrium (*p* > 0.05). Regarding absolute and relative allelic frequencies, we observed a significant predominance of allele I in the *ACE* I/D polymorphism, with a frequency of 63.6% (χ2 with Yates correction = 13.72 and *p* = 0.0002, adjusted *p*-value: 0.005). The effect size of allele I was calculated as high (0.58). Although the *CK* MM Ncol polymorphism did not show a significant difference for allele A (58.8%, χ2 with Yates correction = 3.17 and *p* = 0.07, adjusted *p*-value: 1.75), the effect size of allele A was considered moderate (0.28).

Table 3 explores the relationship between the genetic variants in the *ACTN3* R577X, *ACE* I/D, and *CK* MM A/G Ncol loci and the mean race times, along with standard deviations, in minutes. No statistically meaningful differences were observed (*p* > 0.05) were observed among the RR genotype of the *ACTN3* polymorphism (965 ± 138.8 min), the II genotype of the *ACE* I/D gene (1034.4 ± 142.2 min), and the GG genotype of the *CK* MM A/G Ncol gene (1028.7 ± 218.9 min), despite these genotypes showing lower mean values compared to others.

The correlations between the athletes’ running time in minutes (1096.8 ± 174.22) and the minimum and maximum values of minutes (778–1309) with the mean and standard deviation of the electrochemical variables and acid-base disturbance are presented in Table 4. It can be observed that there was no significant correlation between the electrochemical variables and the running time in minutes, except for the pre-race Ca^2+^ (1.25 ± 0.09 mmol/L) and the post-race Ca^2+^ (1.13 ± 0.04 mmol/L), which showed a positive correlation (Rs = 0.35 for *p* = 0.01). This suggests that the greater the loss of Ca^2+^, the shorter the running time.

**Figure 1:**
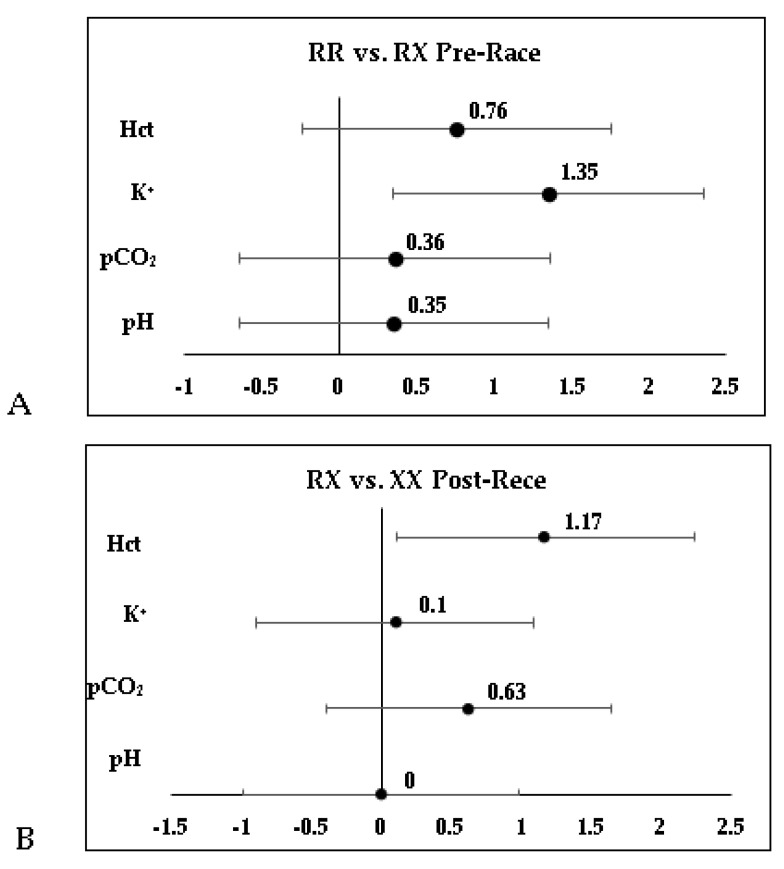
Comparison of biochemical variables between ACTN3 R577X genotypes pre- and post-105 km mountain ultramarathon. (**A**) RR vs. RX pre-race; (**B**) RX vs. XX post-race.

Figure 1A (RR vs. RX Pre-Race) suggests that athletes possessing the RR genotype exhibited markedly elevated levels of K+ (5.92 ± 0.56 mmol/L) compared to RX (5.08 ± 0.60 mmol/L), with *p* = 0.01, as reflected by a Hedges’ g of 1.35. Other variables, such as Hct (0.76), pCO_2_ (0.36), and pH (0.35), did not show statistical significance (CIs included zero). Figure 1B (RX vs. XX Post-Race) highlights those athletes with the XX genotype had higher Hct levels (48.20 ± 4.09%) compared to RX (44.25 ± 2.80%), with *p* = 0.03 and a Hedges’ g of 1.17, suggesting greater oxygenation capacity. Other variables showed no significant differences (*p* > 0.05).

**Figure 2:**
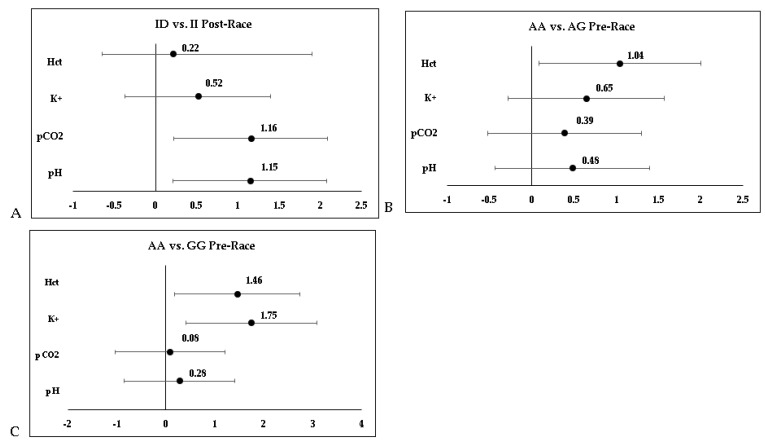
Comparison of biochemical variables between ACE I/D and CK MM A/G NcoI polymorphism genotypes pre- and post-105 km mountain ultramarathon. (**A**) ID vs. II post-race; (**B**) AA vs. AG pre-race; (**C**) AA vs. GG pre-race.

Figure 2A (ID vs. II Post-Race). Compares the ID and II genotypes post-race for the biochemical variable’s pH and pCO_2_. The results show that athletes with the II genotype had a significantly higher pH (7.45 ± 0.04) compared to those with the ID genotype (7.41 ± 0.03), with *p* = 0.02, suggesting reduced susceptibility to metabolic acidosis. For pCO_2_, athletes with the II genotype exhibited lower values (32 ± 1.63 mmHg) compared to ID (33.93 ± 1.59 mmHg), with *p* = 0.01, indicating a possible difference in CO_2_ regulation during physical exertion. Figure 2B (AA vs. AG Pre-Race. Shows the comparison of Hct values between ID and II genotypes post-race. The data indicate that athletes with the II genotype presented significantly higher Hct levels (47.45 ± 2.58%) compared to ID (45 ± 1.53%), with *p* = 0.03. This suggests a greater oxygen transport capacity in the II genotype group, potentially reflecting superior physiological adaptation post-race. Figure 2C (AA vs. GG Pre-Race). Compares the AA and GG genotypes for K^+^ and Hct variables post-race. Athletes with the GG genotype showed higher K^+^ levels (5.98 ± 0.67 mmol/L) compared to AA (5.01 ± 0.40 mmol/L), with *p* = 0.01, indicating potential differences in potassium homeostasis. Hct values were also significantly higher in the GG group (47.75 ± 2.06%) compared to AA (45 ± 1.53%), with *p* = 0.03, suggesting a potential advantage in oxygen transport capacity in the GG genotype group.

## 4. Discussion

To reinforce, our study investigated the impact of the *ACTN3* R577X, *ACE* I/D, and *CK* MM A/G NcoI polymorphisms on electrochemical characteristics in 105 km mountain runners. Our findings partially supported the hypothesis, demonstrating that certain genotypes of the *ACTN3* R577X and *ACE* I/D polymorphisms were significantly related to electrochemical parameters, while the *CK* MM A/G NcoI polymorphism showed no consistent correlations.

An ultramarathon, lasting over 10 h, presents significant physiological challenges compared to a 42.195 km marathon [2]. Due to these demands, athletes are susceptible to early fatigue from prolonged concentric and eccentric actions during ascents and descents [16]. Our findings are aligned with the study by Tiller and Millet [17], which indicates that muscle damage is a key limiting factor for performance, exacerbated by mechanical demands and hindering recovery during races. Additionally, as noted by Sabater-Pastor et al. [18], optimizing VO_2_max and speed is essential to delaying fatigue and enhancing performance in prolonged endurance events. This perspective is complemented by Delhaye et al. [19] study, which demonstrated that the progressive decline in force and velocity parameters during long races underscores the measurable impact of fatigue on performance, reinforcing the importance of mitigating fatigue effects to maintain performance.

This understanding of the influence of fatigue prompts consideration of how genetic factors contribute to endurance capacity and performance. In our study, the athletes exhibited intermediate genotypes associated with skeletal muscle strength and endurance, which are essential traits for success in endurance sports [20]. Notably, the overall winner presented the RX, ID, and AG genotypes, suggesting that these genetic profiles may contribute to energy conservation and sustained performance during a 105 km ultramarathon [16].

However, these findings contrast with previous studies that did not find significant associations between these polymorphisms and performance in long-distance races. Studies by Papadimitriou et al. [21] and Chen et al. [22] found no significant correlations between the polymorphisms in the *ACTN3* R577X, *ACE* I/D, and *CK* MM A/G NcoI genes and endurance performance. Such discrepancies may stem from methodological limitations, including differences in performance phenotype measurements and small sample sizes, which can affect the reliability of results [23].

When we analysed the allelic distributions of the *ACTN3* R577X polymorphism, the R and X alleles did not show significant percentage differences. These results should be interpreted with caution, as they may be affected by multiple elements, including recovery, nutrition, psychological skills, and adaptations to heat and altitude [24]. In our study, the allele distribution for the *ACE* I/D variant indicated a predominance of the I allele, which has been linked to enhanced endurance. Yang et al. [25] reported that national-level soccer athletes had a frequency of 65.8% for the I allele. In another study, Eroğlu et al. [26] showed that Turkish athletes (short, medium and long-distance running) had 52% I alleles. Both studies agree with our study.

Regarding the allele distribution in the *CK* MM A/G Ncol variant, the predominance of the A allele suggests a genetic predisposition toward endurance sports among the sampled athletes. Our findings are consistent with those of Chen et al. [22], who observed that the G allele is more prevalent in athletes focused on power activities and may serve as a safeguard against muscle degradation under physical stress. The assessment of running performance suggested that individuals with the RR genotype in the ACTN3 gene had shorter running times compared to those with the RX genotype. This supports the notion that the RR genotype, characterized by higher levels of *ACTN3*, contributes to enhanced lean mass, strength, and muscle power, improving muscle contraction and adaptability to training and competition [27].

Belli et al. [28] investigated the performance of ultra-runners and reported no significant differences in median race times between genotype groups, indicating that genotype alone may not be a strong predictor of performance. Similarly, Chiu et al. [2] found a trend suggesting better performance among runners with the D polymorphism, although the evidence was not conclusive (*p* = 0.036). Other studies, such as those by Papadimitriou et al. [21], demonstrated an absence of a definitive link among the *ACTN3* R577X, *ACE* I/D, and *CK* MM A/G Ncol genotypes and athletic performance across different distances. While these findings offer valuable context, our study adds to the field by focusing on the electrochemical and acid-base shifts observed during ultra-endurance events. We confirmed that ultra-endurance races significantly impact physiological variables, including pH, electrolytes (Na^+^, K^+^), and decreases in Hct, Glu, Lac, Ca^2+^, pCO_2_, and HCO_3_^−^ levels. This underscores the importance of precise monitoring to optimize athlete performance and recovery [29,30].

In our study, we found a significant correlation between the loss of Ca^2+^ and shorter running times, with the top four finishers showing an average decrease of 43.8% (SD = 13.8%) in Ca^2+^ levels. This suggests that greater Ca^2+^ depletion may be linked to better performance in ultra-endurance events. Our results are consistent with earlier observations by Kłapcińska et al. [31], which documented a reduction in calcium ion (Ca^2+^) levels associated with the total distance covered during extended running. The progressive decline in Ca^2+^ levels could be attributed to acute respiratory alkalosis, which increases pH levels [32]. Endurance training may enhance the body’s capacity to buffer acid-base imbalances without affecting performance, as noted by Barbieri et al. [33], supporting the physiological adaptations observed in our study.

Our data indicate that Hct values were the least affected in athletes with the XX genotype of the *ACTN3* gene compared to those with the RX genotype, suggesting a potential advantage in maintaining stable Hct levels during endurance events. This stability may contribute to improved blood viscosity and oxygen transport, factors crucial for sustaining performance and overall health [34]. The observed resistance to haemolysis and oxidative damage in XX genotype athletes supports the notion that this genotype may modulate vascular function and enhance the ability to resist fatigue [35,36].

When analyzing pH levels, our results showed that genotype II of the *ACE* gene showed pH modulation. Athletes with genotype II had higher pH levels than genotype ID, reflecting directly on race time, as athletes with genotype II had shorter race times than genotype ID. Lower pH levels cause metabolic acidosis, impairing muscle function and compromise performance [30]. Then, it is essential to maintain the normal range of pH to ensure the continuity of physiological processes, such as protein synthesis, intermediary glucose metabolism, cell growth and reproduction [37].

When comparing the II genotype with the ID genotype, our study found that athletes with the II genotype exhibited lower pCO_2_ values and higher pH levels post-race. This suggests a potential association of the ACE II genotype with the development of respiratory alkalosis during intense exertion. The association of the II genotype with lower *ACE* levels may enhance endothelium-dependent vasodilation, which supports a higher acid-base buffering capacity and contributes to improved performance and recovery [22]. High pCO_2_ levels, on the other hand, were associated with increased metabolic acidosis, reducing the athlete’s ability to sustain performance [38].

Despite limitations, such as controlling food intake and mineral salt supplementation during the race, which may have contributed to changes in markers and electrochemical disturbances, this does not compromise the study’s validity. It demonstrated the relationship between the studied polymorphisms, electrochemical variables and acid-base disturbances. Although the sample size of 22 athletes may be considered modest, it was carefully justified based on a power analysis conducted using GPower software. With a standardized difference of 0.7, a significance level (α) of 0.05, and a statistical power of 0.8, it was determined that a sample of 19 participants would be sufficient to detect significant differences in the variables investigated. Thus, the sample of 22 athletes not only meets but exceeds this recommended number, providing greater reliability to the results.

Moreover, by selecting an elite group of Brazilian mountain ultramarathon runners, homogeneity was ensured, minimizing environmental influences and external phenotypic variations. This specific group allows for the identification of associations between genetic variants and physiological responses of interest, such as electrochemical variables and acid-base balance, without interference from diverse external factors. This balanced approach between statistical power and the biological specificity of the group makes the sample size adequate and sufficient to investigate the effects of ACTN3 R577X, ACE I/D, and CK MM A/G NcoI polymorphisms in a high-performance group.

Furthermore, considering the literature reviewed by the authors of this study, the current research is considered an innovative approach to analysing high-performance athletes. Thus, the interplay between genetic factors and athletic performance underscores the importance of acknowledging biological individuality in the evaluation and preparation of 105 km mountain ultramarathon athletes. To further clarify the research direction proposed by this study, future investigations should examine these associations in a broader sample of runners, including female athletes, genetic comparisons, electrochemical analyses, and acid-base disturbances among athletes covering the same distance at varying altitudes. This can enhance our understanding of the genetic impact on adaptability and performance in mountain ultramarathons. Finally, from a practical perspective, the genetic profile combined with biochemical characteristics could form a robust predictive package, enabling athletes and their teams to better optimize training processes.

## 5. Conclusions

The results of this study indicate that mountain ultramarathon athletes who competed at a 105 km distance displayed an intermediate phenotype concerning strength, power, and endurance. This suggests that various genetic factors may influence ultra-endurance sports. We observed a correlation between the loss of calcium and race time, suggesting that greater calcium loss leads to shorter race times. This is intriguing as we did not control calcium supplementation during the event. Athletes with the XX genotype appear less affected by hematocrit modulation, which may indicate better blood oxygenation than those with the RX genotype.

Additionally, athletes carrying the II genotype of the *ACE* gene showed higher pH levels and lower pCO_2_ values post-race. This suggests they may have experienced respiratory alkalosis during the event, potentially reducing their susceptibility to fatigue. These findings highlight the substantial impact of the R577X variant within the *ACTN3* gene and the *ACE* insertion/deletion polymorphism on the modulation of electrochemical parameters and acid-base balance in well-trained mountain ultramarathon athletes who completed a 105 km race.

## Figures and Tables

**Table 1:** Primers used, as well as their respective gene accession numbers.

Gene	Primer Forward	Primer Reverse	Accession Number
*ACTN3*	5′-CTGTTGCCTGTGGTAAGTGGG-3′	5′-TGGTCACAGTATGCAGGAGGG-3′	89
*ACE*	5′-TGGGACCACAGCGCCCGCCACTAC-3′	5′-CTGGAGACCACTCCCATCCTTTCT-3′	1636
*ACE*	5′-TTTGAGACGGAGTCTCGCTC-3′	5′-GATGTGGCCATCACATTCGTCAGAT-3′	1636
*CK*MM	5′-GTGCGGTGGACACAGCTGCCG-3′	5-CAGCTTGGTCAAAGACATTGAGG-3	1158

**Table 2:** Genotypic and allelic distribution of the polymorphisms of the genes *ACTN3* R577X, *ACE* I/D and *CK* MM A/G Ncol in 22 mountain ultramarathon athletes.

Polymorphism	Genotype	n	Genotypic Frequency (%)	*p*-Value	Adjusted *p*-Value (Bonferroni)	Cramer’s V	Allele	n	Allelic Frequency (%)	*p*-Value	Adjusted *p*-Value (Bonferroni)	Cramer’s V
*ACTN3* R577X	RR	5	22.7	0.0001 *	0.001	1.12	R	22	50	ns	ns	-
	RX	12	54.5				X	22	50			
	XX	5	22.7									
*ACE* I/D	DD	1	4.5	0.0001 *	0.001	0.98	D	16	36.4	0.0002 *	0.005	0.58
	ID	14	63.6				I	28	63.6			
	II	7	31.8									
*CK* MM A/G Ncol	AA	7	31.8	0.007 *	0.07	0.62	A	19	43.2	0.07	1.75	0.28
	AG	11	50				G	25	56.8			
	GG	4	18.2									

* *p* ≤ 0.05, RR = functional/functional, RX = functional/non-functional, XX = non-functional/non-functional, DD = homozygous for the deletion allele, ID = heterozygous with one insertion and one deletion allele, II = homozygous for the insertion allele, GG = restriction/restriction, AG = insertion/restriction, AA = insertion/insertion. Note: *p* ≤ 0.05 indicates statistical significance, suggesting that the observed distribution of genotypes and alleles is unlikely to occur by chance. The *p*-values presented in the table reflect the statistical analysis of the genotypic and allelic distribution of the polymorphisms.

**Table 3:** Evidence times were obtained concerning the genotypes of polymorphisms (R577X, ID and NcoI) of alpha-actinin-3 (ACTN3), angiotensin-converting enzyme (ACE), and muscle-specific creatine kinase (CK MM) genes of the study sample (*n* = 22).

Polymorphisms	Genetic Type (*n*)	Time (min)	*p* Significance	Hedges’ g	95% CI
*ACTN3* R577X	RR (*n* = 5) vs. RX (*n* = 12)	965 ± 138.8–1135.7 ± 171.7	0.06	0.99	−0.05–2.04
	RR (*n* = 5) vs. XX (*n* = 5)	965 ± 138.8–1135.2 ± 175.5	0.12	0.97	−0.23–2.17
	RX (*n* = 12) vs. XX (*n* = 5)	1135.7 ± 171.7–1135.2 ± 175.5	0.99	0.00	−1.12–1.12
*ACE* ID	ID (*n* = 14) vs. II (*n* = 7)	1125.5 ± 191.3–1034.4 ± 142.2	0.28	−0.49	−0.39–1.38
*CK* MM AG NcoI	AA (*n* = 7) vs. AG (*n* = 11)	1124.4 ± 132.6–1104 ± 190.6	0.88	−0.11	−1.02–0.79
	AA (*n* = 7) vs. GG (*n* = 4)	1124.4 ± 132.6–1028.7 ± 218.9	0.38	0.53	−0.62–1.67
	AG (*n* = 11) vs. GG (*n* = 4)	1104 ± 190.6–1028.7 ± 2018.9	0.52	0.36	−0.73–1.44

**Table 4:** Correlation between race time and electrochemical variables and acid-base disorder for the study sample (*n* = 22).

Variables	Reference Values	Pre-Race	Post-Race	Running Time (Rs)	*p* Significance
pH	7.38–7.44	7.42 ± 0.02	7.42 ± 0.03	0.01	0.72
pCO_2_ (mmHg)	38–42	40.05 ± 3.09	33.36 ± 1.81	−0.15	0.49
pO_2_ (mmHg)	80–100	67.64 ± 1.28	66.45 ± 9.42	−0.22	0.16
Na^+^ (mmol/L)	136–145	141.82 ± 1.74	140.04 ± 3.74	0.10	0.38
K^+^ (mmol/L)	3.5–5.0	5.39 ± 0.67	4.35 ± 0.35	0.20	0.15
Ca^2+^ (mmol/L)	1.12–1.23	1.25 ± 0.09	1.13 ± 0.04	0.35	0.01 *
Glu (mg/dL)	70–99	94.86 ± 9.84	98.31 ± 18.10	−0.08	0.13
Lac (mmol/L)	0.7–2.1	2.26 ± 0.86	2.78 ± 1.02	0.15	0.67
Hct (%)	42–50%	46.73 ± 2.43	45.36 ± 3.34	0.38	0.12
HCO_3_^−^ (mmol/L)	23–26	26.16 ± 1.77	21.77 ± 1.52	−0.06	0.89

Hydrogen ion concentration (pH), partial CO_2_ pressure (pCO_2_), partial O_2_ pressure (pO_2_), Na^+^ (Sodium), K^+^ (Potassium), Ca^2+^ (Calcium), Glu (glucose), Lac (lactate), hematocrit (Hct) and HCO_3_^−^ (Sodium Bicarbonate), * *p* ≤ 0.05.

## Data Availability

Data are not publicly available due to privacy restrictions.
